# Cost-Effective Edge Server Placement in Wireless Metropolitan Area Networks

**DOI:** 10.3390/s19010032

**Published:** 2018-12-21

**Authors:** Feng Zeng, Yongzheng Ren, Xiaoheng Deng, Wenjia Li

**Affiliations:** 1 School of Software, Central South University, Changsha 410083, China; fengzeng@csu.edu.cn (F.Z.); forstudy@csu.edu.cn (Y.R.); 2 Department of Computer Science, New York Institute of Technology, New York, NY 10023, USA

**Keywords:** edge server placement, mobile edge computing, minimum dominating set, simulated annealing

## Abstract

Remote clouds are gradually unable to achieve ultra-low latency to meet the requirements of mobile users because of the intolerable long distance between remote clouds and mobile users and the network congestion caused by the tremendous number of users. Mobile edge computing, a new paradigm, has been proposed to mitigate aforementioned effects. Existing studies mostly assume the edge servers have been deployed properly and they just pay attention to how to minimize the delay between edge servers and mobile users. In this paper, considering the practical environment, we investigate how to deploy edge servers effectively and economically in wireless metropolitan area networks. Thus, we address the problem of minimizing the number of edge servers while ensuring some QoS requirements. Aiming at more consistence with a generalized condition, we extend the definition of the dominating set, and transform the addressed problem into the minimum dominating set problem in graph theory. In addition, two conditions are considered for the capacities of edge servers: one is that the capacities of edge servers can be configured on demand, and the other is that all the edge servers have the same capacities. For the on-demand condition, a greedy based algorithm is proposed to find the solution, and the key idea is to iteratively choose nodes that can connect as many other nodes as possible under the delay, degree and cluster size constraints. Furthermore, a simulated annealing based approach is given for global optimization. For the second condition, a greedy based algorithm is also proposed to satisfy the capacity constraint of edge servers and minimize the number of edge servers simultaneously. The simulation results show that the proposed algorithms are feasible.

## 1. Introduction

In recent years, portable devices such as mobile phones, tablets and laptops have dominated most people’s daily lives due to their convenience and powerful functions. Unfortunately, the performance of these portable devices appears poor, as the applications such as face recognition, natural language process and augmented reality are becoming more and more compute-intensive. Moreover, mobile devices themselves are restricted by their own battery lives, computation capacities and sizes. Mobile cloud computing (MCC) [1] has been widely used to cross the barrier mentioned above, whose main idea is that offloading the compute-intensive tasks of mobile devices to remote clouds with substantial computation resources rather than executing them locally. Although MCC is fruitful, it incurs new problems. On the one hand, the distance between mobile devices and remote clouds might be too far to tolerate. On the other hand, a tremendous number of mobile devices connect to remote clouds, and compete for computational resources with each other, which can lead to network congestion. Both sides will make the user experience terrible, since most applications are not only compute-intensive but also latency-sensitive. A promising approach to overcome the shortages of MCC is to bring computation and storage resources to the edge of network, which means enabling the edge of network to be the optional destinations for task offloading, and Mobile Edge Computing (MEC) [2], cloudlet computing [3], and fog computing [4] are such technologies proposed to utilize the edge of the network to mitigate the burdens of remote clouds and reduce delay [5,6]. In this paper, for convenience and consistency, we refer to those devices with rich resources (e.g., a MEC server, a fog node or a cloudlet) as edge servers while ignoring specific differences among them [7]. With the help of edge servers, mobile devices can offload all or part of their assignments to them without caring about what will happen, and they just need to receive the execution results [8], which augments the performance of mobile devices and relieves the burdens of remote clouds greatly.

Despite the wonderful advantages of edge servers, to a large extent, where and how to deploy them has been neglected. Most studies assumed that the edge servers they need have been properly deployed and merely concentrate on the offloading and resource allocation strategies [9,10]. However, without optimal edge server placement strategies, these jobs may be unrealistic because all the services offered by MEC are based on the reality that edge servers has been deployed and can be harnessed easily. In addition, edge server placement strategies are related with not only user experience but also the cost of MEC service vendors. Therefore, it is of significant importance to investigate the edge server placement problems as to construct a solid foundation for the other works in MEC.

There exist a few studies on the edge server placement problems [11,12], and most researchers overlook the problem of how to minimize the number of edge servers while ensuring delay constraint, which is vital to user experience and service providers. Since the budgets of MEC service vendors are always limited, it is impossible to deploy edge servers everywhere and it is encouraged to reduce cost as much as possible instead. The cost of deploying edge servers is mainly related to two factors [13], which are site rentals and computation demands. The former factor means that the more locations are selected to deploy edge servers, the higher is the cost. The latter factor tells us that the greater is the computation demand, the greater is the number of edge servers, which will result in higher cost. In practice, at the beginning of a project, the vendors often do not have enough funds to support the best service, but just to meet the basic needs of users for reducing cost as much as possible. For example, a MEC service provider is planning to deploy the MEC systems to serve all the population in a city. Regrettably, the capital of this MEC service provider is restricted, which means that it is impossible to deploy edge servers everywhere. The urgent matter is to economize while ensuring the basic requirements of users, such as access delay. Redesigning a wholly new scheme is always expensive and impractical. In addition, edge server deployment scheme is directly connected with the business models, and different deployment schemes will result in different business benefits. Fortunately, we can utilize the legacy of existing technologies to deploy MEC service effectively and economically. Wireless metropolitan area networks (WMAN), consisting of a large number of wireless access points (APs), has attracted attentions of academic areas naturally due to its available infrastructure [14]. It is certain that deploying edge servers on existing APs can save more funds than deploying them on new places.

In this paper, we investigate how to make the greatest use of the legacy of existing WMAN to deploy edge servers efficiently and economically. It is the first time to address the problem of minimizing the number of edge servers while ensuring access delay requirement in a WMAN context. There are two main challenges in this problem: one is how to choose the fewer APs co-located with an edge server to provide all users with good service, and the other is how to properly assign the tasks to edge servers. In response to these challenges, we divide the WMAN into clusters, and the cluster heads are co-located with edge servers. In a cluster, all members offload the tasks to the cluster head. We extend the definition of dominating set, and transform the addressed problem into the minimum dominating set problem in graph theory. According to different conditions, we propose the greedy and simulated annealing based algorithms to find the optimal solutions.

The remaining parts of this paper are organized as follows. Related works are reviewed in Section 2. The network model, Integer Linear Programming (ILP) formulation and the definitions of aforementioned two categories of problems are presented in Section 3. Two heuristic algorithms are proposed for the first kind of problem in Section 4. Then, a greedy heuristic approach is introduced for the second kind of problem in Section 5. An example of the solutions of these two problems is given in Section 6. Simulation results are provided to evaluate the performance of the proposed algorithms in Section 7. Section 8 concludes the paper and future works.

## 2. Related Work

For the promising vision of MEC, research interest in MEC has grown dramatically [15]. Quite a few papers survey f existing research works, and present current challenges, future directions and the recently-emerged open issues [16,17,18,19,20,21,22]. One of the most important functions of MEC is to provide the computation for offloaded tasks from the edge servers, so that the mobile devices can save energy and speed up the process of tasks. In the literature, there exists a large quantity of research regarding computing offloading. This problem could be divided into several sub-problems, such as whether to offload, what should be offloaded, what the order of offloaded task should be, etc. If the local devices are able to conduct the tasks, just execute them locally. Due to the size of the data that need to be transferred and the latency caused by the transmitting process, whether to offload full tasks or to offload part of them should be considered. Moreover, when the offloading tasks are given, the sequence of these tasks is also vital to ensure that it returns the correct results. Zhang et al. [23] proposed an efficient code partition algorithm to help make decisions for offloading. Taking the dependency of offloaded components into consideration, Mahmoodi et al. [24] made decisions for offloading according to not only components needed to be offloaded, but also the scheduling order of these components. Mach et al. [25] surveyed the current research work and classified them into three types: decision on computing offloading, allocation of computing and mobility management. Although there are several reports on these three hot topics, the edge server placement problem has been neglected, while it is significant as well.

To date, and to the best of our knowledge, there are only a few works focused on the edge server placement problem. Satyanarayanan et al. [26] showed that application placement had great impact on user experience, and gave evidence that moving applications closer would indeed improve user experience. Xu et al. [14] first formulated a novel capacitated cloudlet placement problem, which placed *K* cloudlets to strategic locations to minimize the average cloudlet access delay between the mobile users and the cloudlets in WMAN, and showed that the problem is NP-hard. Then, they proposed serval efficient algorithms to solve it. Considering the redundant sorting process of Xu’s algorithms, the authors of [27] proposed a new heuristic algorithm and a Particle Swarm Optimization (PSO) algorithm which outperformed the existed algorithms. Jia et al. [28] presented an efficient algorithm to deploy a limited number of cloudlets in a WMAN while balancing their workload. Meng et al. [29] raised an algorithm based on the snapshots of the network to place the edge servers in permanent locations and an online job routing algorithm to minimize the communication delay. Taking the cost of deploying edge servers into consideration, Fan et al. [30] proposed a cost aware cloudlet placement strategy to optimize the tradeoff between the deployment cost and end-to-end delay. In view of the mobility of mobile users, Xiang et al. [31] introduced movable cloudlets and a novel cloudlet placement method for mobile applications over GPS big data. Considering the convergence of Fog Computing and IoT, Naas et al. [32] formulated the data placement problem as a generalized assignment problem and a heuristic approach, namely iFogStor, which is devised to reduce latency. Some other related works focus on virtual machine replica copies (VRCs) placement problem (e.g., [33,34]), where several optimal VRCs strategies are proposed to minimize the average response time. It is worth noting that European Telecommunications Standards Institute (ETSI) has put hands to study the compatibility of MEC with current technologies such as 4G [35], which is consistent with our idea of utilizing the legacy of existing technologies. Due to similarities to edge server deployment, SDN controller placement is a related problem that has attracted more and more attention as well. Given the IoT networks, authors of [36] applied Software-Defined Networking (SDN) techniques to provide flexible and programmable management for cloudlet placement and proposed a ranking-based near-optimal placement algorithm. Heller et al. [37] first proposed the SDN controller placement problem to investigate where and how many SDN controllers should be deployed. Taking the controller capacity into consideration, Yao et al. [38] first defined the capacitated controller placement problem and devised two phase algorithm to solve it. Different with the problem we focus on, SDN controller placement emphasizes flexible and programmable management of network, and the related methods cannot be used in the edge server placement in WMAN.

The existing studies have explored how to utilize the legacy of WMAN to deploy edge servers or apply SDN technologies to help the deployment of edge servers, but most only pay attention to minimizing the average delay between edge servers and mobile users, and there are limited studies that focus on minimizing the number of edge servers while ensuring the access delay requirement. Different from existing studies, in this paper, we investigate the edge server placement problem in an economic perspective. Our objective is to reduce cost as much as possible and meet the requirements of mobile users at the same time.

## 3. System Model

In this section, we first describe the details of the network model. Then, the precise definition of the edge server placement problem is given by formulating it as an ILP.

### 3.1. Network Model

**Figure 1:**
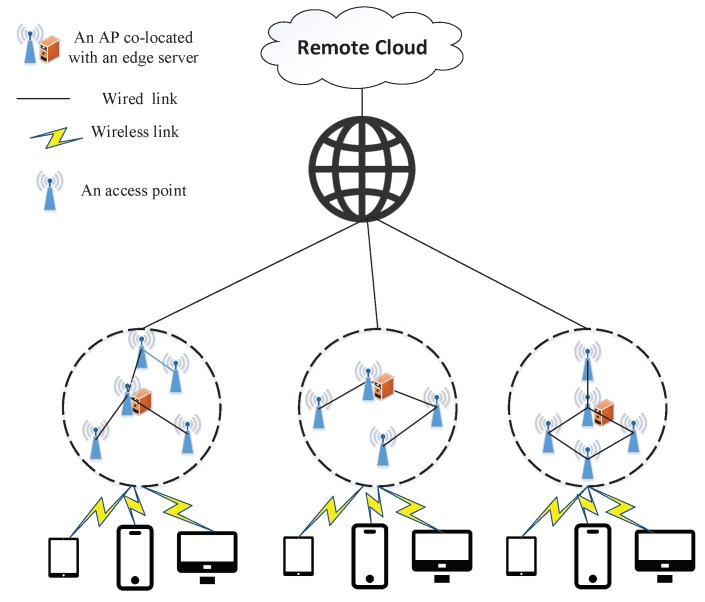
Mobile edge computing architecture.

The architecture of MEC is shown in Figure 1, consisting of remote cloud, Internet, APs and edge servers. To utilize the legacy of WMAN, we divide the WMAN into disjoint clusters (the circles made up of dashed lines in Figure 1), which comprise different numbers of APs, and the edge servers are placed at the cluster heads. Generally speaking, the sizes of the clusters are limited and different. For any AP with computation tasks needed to be offloaded, the edge server within the cluster is the first choice, and the edge servers are connected by the Internet to prevent failures, thus the tasks of an AP can be offloaded to heads of other clusters if its own cluster head is in trouble or overloaded. Furthermore, the remote cloud is another choice for the offloaded tasks.

We consider the problem of edge server placement in the context of WMAN. A WMAN can be denoted by a connected undirected graph G=(V,E) where *V* is the set of APs and *E* is the set of links between APs. There is an edge (u,v)∈E, if and only if APs *u* (u∈V) and *v* (v∈V) are connected by a communication link. It is denoted that n=|V| is the number of APs, m=|E| is the number of links between APs, and Neighbor(i) is the set of node *i*’s neighbors in graph *G*. Each AP could be selected as the location of an edge server. That is, the edge servers are co-located with part of APs instead of selecting some other places that might cause not only additional delay but also additional cost. To be more practical, we divide the Edge Server Placement Problem (ESPP) into two categories. The first one is that the capacities of edge servers can be distinct, and we customize them as on-demand edge servers, which implies the capacity of an edge server is determined by the amount of computation tasks offloaded to it. We call this kind of problem Edge Server Placement Problem withOut Constraint (ESPPOC) because it seems that there is no capacity constraint on edge servers. The second one is that all capacities of edge servers must be identical and limited, which is in contrast to the first one. Due to the limited capacities of edge servers, we refer to it as Edge Server Placement Problem With Constraint (ESPPWC). We discuss these two problems separately. Before the detailed analysis of them, we first describe the basic concepts of our network model. For simplicity, we assume that each hop between two APs has the same access latency, even though the physical distances between them may be different, since the differences of physical distances among APs are negligible compared to the physical distances between APs and a remote cloud. We denote the distance between APs *u* and *v* as d(u,v), which is the minimum number of hops between them, and we can obtain it by computing the shortest path between them. Generally speaking, the delay between two APs is proportional to the distance between them. That is, denoting D(u,v) as the delay between APs *u* and *v*, D(u,v) is proportional to d(u,v). Consequently, in the following sections, d(u,v) can represent the access delay requirement of mobile users.

### 3.2. Problem Formulation

In this paper, we focus on minimizing the number of edge servers while ensuring the latency constraint. The definition of the problem is described as follows. Given a WMAN G=(V,E) and a delay upper bound $D_{max}$, our objective is to find the minimum subset K⊂V, which could connect all APs in *V* with the $D_{max}$ constraint. We define a binary variable $x_{i}$ (i∈V) to represent whether an edge server is deployed at AP *i* (i.e., $x_{i}=1$) or not (i.e., $x_{i}=0$), and another binary variable $y_{\left(i,j\right)}$ (j∈K) to denote whether the workload of AP *i* is assigned to edge server *j* (i.e., $y_{\left(i,j\right)}=1$) or not (i.e., $y_{\left(i,j\right)}=0$). To the nodes assigned with the same edge server *j* (j∈K), they and *j* together form a cluster, and node *j* is the cluster head co-located with an edge server. Then, we use c(j) and r(i) to denote the capacity of edge server *j* and computation demand of AP *i*, respectively. Generally speaking, the computation demand of a AP is proportional to its traffic load, which can be obtained from historical data provided by the service operator. In the short term, the movement of mobile users may impact the traffic load of APs. However, in the long run, the network traffic distribution remains relatively stable. Consequently, from historical data, we can give r(i) a reasonable value reflecting the real situation. Recall that d(u,v) is the minimum hops between APs *u* and *v*, and the delay D(u,v) between APs *u* and *v* is proportional to d(u,v). Thus, the delay constraint $D_{max}$ can be converted to the distance constraint $d_{max}$. In addition to delay constraint, we also consider the constraints of node degree and cluster size, since too many neighbors for a node or too many nodes in a cluster will certainly make the network QoS worse. We define deg(v) as the degree of *v* in its cluster, deg(v) must be less than $D_{q}$ for any given node *v*, and the number of APs assigned to the same edge server cannot be greater than $S_{q}$. That is, $S_{q}$ is the upper bound of cluster size, which may avoid network congestion in the cluster. It can be formulated as an ILP. Our objective function is formulated as follows.

$$min\sum_{i\in V}x_{i}$$

Subject to

(1) $$\sum_{i\in V}y_{\left(i,j\right)}\cdot r\left(i\right)\leq c\left(j\right),\forall j\in K$$

(2) $$\sum_{j\in K}y_{\left(i,j\right)}\cdot d\left(i,j\right)\leq d_{max},\forall i\in V$$

(3) $$\sum_{j\in K}y_{\left(i,j\right)}=1,\forall i\in V$$

(4) $$deg\left(v_{i}\right)\leq D{}_{q},\forall i\in V$$

(5) $$\sum_{i\in V}y_{\left(i,j\right)}\leq S_{q},\forall j\in K$$

(6) $$x_{i}\in \left\{0,1\right\},\forall i\in V$$

(7) $$y_{\left(i,j\right)}\in \left\{0,1\right\},\forall i\in V,j\in K$$

(8) c(j)>0,∀j∈K

(9) r(i)≥0,∀i∈V

(10) $$deg\left(i\right)=|Neighbor\left(i\right)\cap \left\{j\;|\;y_{\left(i,k\right)}=y_{\left(j,k\right)}=1,\forall k\in K\right\}|,\forall i\in V$$

The constraint in Equation (1) ensures that each edge server has enough capacity to process all the computation tasks offloaded to it, and the inequality in Equation (2) ensures that the distance between each AP and the corresponding edge server is no more than the distance upper bound as to satisfy the access delay requirement. The inequality in Equation (4) is the degree limitation and the inequality in Equation (5) represents the cluster size constraint. The inequality in Equations (2), (4) and (5) are considered for the requirements of network QoS. Equation (3) denotes that each AP can only be assigned to only one edge server. The inequality in Equation (1) represents the computation demand constraint, and the inequality in Equation (2) is the delay constraint. If different values are assigned to c(j), it is the definition of ESPPOC problem. Otherwise, it is the ESPPWC problem. In Equation (10), Neighbor(i) is defined as the set of node *i*’s neighbors in graph *G*, and deg(i) is the number of neighbors that are in the same cluster with node *i*. For the poor scalability of ILP, in later sections, our goal is to solve ESPPOC and ESPPWC problem, respectively. We transform them into the minimum dominating set problem in graph theory and appropriate algorithms are devised according to their features. Finally, an example of our solution is given.

## 4. Algorithms for ESPPOC Problem

To solve the ESPPOC problem in a tolerable time, we propose a Greedy based Minimum Extended Dominating Set Algorithm (GMEDS) to get the solution for the placement of edge servers when the delay constraint is given. Then, a Simulated Annealing based Minimum Extended Dominating Set Algorithm (SAMEDS) is devised to prevent the local optimization of GMEDS. The details of these two algorithms are discussed in this section.

### 4.1. Preliminaries

To minimize the number of edge servers while ensuring latency constraint is an analog of the minimum dominating set problem in graph theory is a classical NP-hard problem. Here, we first introduce the basic concepts, then extend the definition of the dominating set, and use it to describe the edge server placement problem. The traditional definition of dominating set is given as follows.

**Definition** **1.** *A dominating set of a graph G=(V,E) is a subset D of V such that every vertex not in D is adjacent to at least one member of D.*

As mentioned above, $D_{max}$ is the upper bound of access delay which users can tolerate, and we assume that the access delay in each hop is the same for convenience. Our objective is to minimize the number of edge servers deployed in a WMAN, if we have a graph *G* representing a WMAN, its minimum dominating set is the edge server placement solution with the delay constraint of $d_{max}=1$. Nonetheless, the minimum dominating set cannot represent the solutions with the delay constraint of $d_{max}>1$, since the distance between the nodes in dominating set and other nodes in Definition 1 is just one hope. To describe the problem with dominating set, we extend the concept of dominating set to meet the requirements of delay constraint more than one hop.

**Definition** **2.** *An i-tier dominating set of a graph G=(V,E) is a subset $D^{i}$ of V such that for every vertex not in $D^{i}$ there exists a link between the vertex and at least one member of $D^{i}$ within i hops.*

From Definition 1, we can know that any node in *G* is either in *D* or one hop away from some node in *D*. In the placement, if edge servers are co-located with the nodes in *D*, AP v∈V has at lease a path within one hop to an edge server. Similarly, from Definition 2, we know that any node in *G* is either in $D^{i}$ or connected with a node in $D^{i}$ within *i* hops. Let the edge servers be co-located with the nodes in $D^{i}$, then $D^{i}$ will be one of edge server placement strategies with the delay constraint *i*. With the help of Definition 2, we can deal with the conditions with the delay constraint more than one hop. Obviously, Definition 2 contains Definition 1, and when *i* is equal to 1, Definition 2 is identical to Definition 1. Let G=(V,E) be the WMAN, our objective is to compute the minimum *i*-tier dominating set $D^{i}$ of *G* according to specific delay constraint *i*.

Recall that the degree of a vertex *v* in a graph *G* is denoted by deg(v), which is the number of edges that connect to vertex *v*. Then, let Neighbor(v) be the vertex set of its neighbors. Corresponding to the *i*-tier dominating set, we denote extNei(v,i) as the set of vertexes which are connected to *v* within *i* hops, and extDeg(v,i) as the number of vertexes in extNei(v,i) (i.e., extDeg(v,i)=|extNei(v,i)|).

### 4.2. Greedy-Based Minimum Extended Dominating Set Algorithm

In this subsection, we use the greedy approach to get the minimum *i*-tier dominating set $D^{i}$ of graph *G*. If $j\subset D^{i}$ or there is a link between any node in $D^{i}$ and *j* within *i* hops, node *j* is said to be “covered”. On the contrary, a node that is not covered is said to be “uncovered”. Obviously, to minimize the number of edge servers is to minimize the number of nodes in $D^{i}$. To this end, for each node *v* in $D^{i}$, we want as many APs as possible to be covered by *v*, which means we should iteratively select an AP with the biggest value of extDeg(v,i) and let it join in $D^{i}$. It is clear that nodes in $D^{i}$ are the cluster heads and the “covered” nodes are the cluster member nodes. Meanwhile, considering the network QoS, the cluster must be adjusted until it is subject to the constraints described in last section. Finally, all nodes will be covered by $D^{i}$, which is the algorithm output representing the strategy of edge server placement. The details of this greedy approach are described in Algorithm 1.

**Algorithm 1** Greedy-based minimum extended dominating set algorithm.**Input:** A graph of the WMAN *G*, the delay constraint i, $D_{q}$ and $S_{q}$; **Output:** Locations of edge servers $D^{i}$ and clusters of APs *C*;
1:U←V; /* *V* is set of nodes in G, *U* is the set of nodes uncovered by $D^{i}$ */2:C←∅; /* *C* is set of the eventual clusters */3:$D^{i}\leftarrow \emptyset ;$ /* $D^{i}$ is the locations of edge servers */4:**while***U***do**5:    $s\leftarrow v_{1};$ /* *s* is initialed by the first node of *U*, namely $v_{1}$ */6:    **for** each v∈U **do**7:        compute the distance between *v* and all other nodes in *U*, denoted by d(v,u)8:        compute the number of the nodes that d(v,u) is no more than i, denoted by extDeg(v,i)9:        **if** extDeg(v,i)>extDeg(s,i) **then**10:           s←v;11:        **end if**12:    **end for**13:    S←s∪extNei(s,i);14:    new_cluster.head←s;15:    new_cluster.nodes←extNei(s,i);16:    **if** there exists a node *v* in *S* whose deg(v) is bigger than $D_{q}$ **then**17:        adjust the cluster until all the nodes satisfy the $D_{q}$ constraint18:    **end if**19:    **if** |S|>$S_{q}$ **then**20:        adjust the cluster until |S| < $S_{q}$21:    **end if**22:    C←C∪new_cluster;23:    $D^{i}\leftarrow D^{i}\cup s$;24:    remove the nodes in *S* from *G*;25:    U←U−S;26:**end while**27:return $D^{i},C$;

As is shown in Algorithm 1, given the access delay constraint *i*, we use the greedy approach to compute the minimum extended dominating set $D^{i}$ of graph *G*. The for-loop from Line 6 to Line 10 selects an AP in *U* with the biggest value of extDeg(v,i) for deploying the edge server, and the cluster *S* is built after the selection process. When the initial cluster is built, we check whether it can satisfy the constraint. If so, this iteration is completed, otherwise we adjust until it can meet the network constraints. It is worth noting that we first adjust the cluster to meet the degree constraint and then make efforts to meet cluster size constraint rather than the reverse order, since adjusting the degree of nodes is bound to influence the cluster size. For the degree constraint, we randomly select a node from the nodes that violate the degree constraint and iteratively delete its neighbor whose degree is the smallest until all the nodes in the cluster satisfy the degree constraint. For the cluster size constraint, we first construct a subgraph $G^{\prime }$ to represent the initial cluster, and then we iteratively delete the nodes which are furthest from the cluster head until the cluster size is no more than $S_{q}$. Eventually, the cluster new_cluster consists of cluster head *s* and the nodes covered by *s*, satisfying the constraint of d(u,s) ≤ *i* (∀u∈S). In addition, the shortest paths between *s* and the nodes covered by *s* are recorded in new_cluster too. As mentioned above, the nodes in new_cluster should offload the computation tasks to the cluster head *s*, which ensures that the distance between all nodes in new_cluster and *s* is no more than *i* hops and all users can access an edge server in a tolerable time.

### 4.3. Simulated Annealing Based Minimum Extended Dominating Set Algorithm

Although GMEDS is able to obtain feasible results, it might get stuck at local optimum solution. Therefore, we adopt a widely used heuristic, namely Simulated Annealing (SA), to convergence a global optimal solution. In this section, we present a brief overview of SA, followed by a detailed discussion on the operations of SAMEDS.

Motivated by the annealing technology in metallurgy, SA was proposed as a metaheuristic to approximate global optimization in a very large search space. It is well known that SA has the ability of jumping out from local optimization to achieve the global optimization, which can be the important supplement to the greedy algorithm mentioned above. There are six primary ingredients in SA: (1) cost function; (2) initial state; (3) neighbourhood generation; (4) cooling schedule; (5) acceptance criteria; and (6) stopping condition. Given an optimization problem, we should first define a proper cost function to effectively evaluate the generated solutions. Then, a initial solution is required to begin the algorithm, and neighbourhood generation methods are needed to help the solution move from one state to another. To jump out from local optimization, the cooling schedule and acceptance criteria should work cooperatively to determine the probability of accepting a new generated solution. In the beginning of SA, a worse solution has a high probability of acceptance. However, as the temperature cools down, SA merely tends to adopt a better solution. Finally, during the SA procedures, the cost function value eventually converges, and the search is terminated if the stopping condition is satisfied. In this paper, we focus on devising an efficient SA method for the ESPPOC problem.

In SA algorithm, we should ensure the processing solutions are the right strategies of edge server placement, which means the solutions must represent an *i*-tier dominating set of the related graph *G*. To construct an *i*-tier dominating set of *G*, we propose the following theorem.

**Theorem** **1.** *Given a graph G=(V,E), a set S, S⊂V, and the tier number i of dominating set of G, the union set of S and the set of nodes uncovered by S within i hops is always an i-tier dominating set of G.*

**Proof** **of** **Theorem** **1.** For each set *S*, S⊂V, let *U* be the set of nodes uncovered by *S* within *i* hops. Each node *v*, v⊂S, can connect at least one member of *S* within *i* hops and for each node *v*, v⊂U, there exists a link between *v* and at least one node in *U*. Thus, for every node not in S∪U, there is a link between the node and at least one member of S∪U within *i* hops, and, according to Definition 2, S∪U is an *i*-tier dominating set of G. ☐

**Figure 2:**
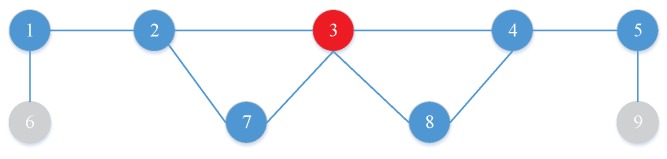
An example of Theorem 1.

An example of Theorem 1 is shown in Figure 2. Given i=2 and a graph G consisting of V={1,2,…,9}, we randomly choose S={3}. In Figure 2, it can be seen that the node set uncovered by *S* within 2 hops is {6,9} and the union set of *S* and {6,9} is {3,6,9} which is a two-tier dominating set of G. Based on Theorem 1, we can generate and evaluate the solutions easily. The detailed discussion of SAMEDS is shown as follows.

#### 4.3.1. Cost Function

Given a graph G=(V,E) and a set *S*, S⊂V, let uncover(S,i) be the number of nodes uncovered by *S* within *i* hops. From Theorem 1, we can know that *S* does not have to be a *i*-tier dominating set, but *S* together with the uncovered nodes by *S* constitutes an *i*-tier dominating set. We define the cost function of *S* as Equation (11). Our objective is to minimize the value of calCost(S,i).

(11) calCost(S,i)=|S|+uncover(S,i)

#### 4.3.2. Initial State

Obviously, *V* is always an instance of *i*-tier dominating set for any given *i*, and calCost(V,i) is equal to the number of nodes in *V* all the time. Therefore, *V* is selected to be the initial solution of SAMEDS.

#### 4.3.3. Neighborhood Generation

There are three ways to move from current state to another state in our algorithm: (1) adding a random node from the set of uncovered nodes to current solution; (2) deleting a random node from current solution; and (3) selecting a node from current solution randomly and replacing it with a random node from uncovered nodes. All of these ways are able to change the calCost(S,i) value of current solution to achieve the state movement in SAMEDS.

#### 4.3.4. Cooling Schedule

We define a cooling factor α for the classic cooling schedule in this paper to control the cooling schedule of SAMEDS. For each temperature, iter iterations are executed to search a solution with good quality and avoid getting stuck in local optimization. As shown from Line 6 to Line 22 in Algorithm 2, the cooling process is accompanied by the decrease of iter, which is designed to fully explore the local optimization at lower temperatures and reduce unnecessary exploration for saving execution time at higher temperatures.

**Algorithm 2** Simulated annealing-based minimum extended dominating set algorithm.**Input:** A graph of the WMAN *G* and the delay constraint i; **Output:** Locations of edge servers, *S*, clusters of APs, *C*;
1:C←∅; /*C is set of the eventual clusters /*2:S←V; /*S is the locations of edge servers */3:$T\leftarrow T_{0}$4:cur_cost←calCost(S,i)5:**while**$T>T_{fin}$**do**6:    **for** k<iter **do**7:        new_S←createNei(S);8:        new_cost←calCost(new_S);9:        Δ←new_cost−cur_cost10:        **if** Δ<0 **then**11:           cur_cost←new_cost12:           S←new_S13:        **else**14:           p←exp(−Δ/T)15:           **if** rand()<=p **then**16:               cur_cost←new_cost17:               S←new_S18:           **end if**19:        **end if**20:        k←k−121:    **end for**22:    T←T∗α23:**end while**24:**for**v∈S**do**25:    cluster.head←v26:    cluster.nodes←extNei(v,i)27:    C←C∪cluster28:    $S^{\prime }\leftarrow v\cup extNei\left(v,i\right)$29:    remove $S^{\prime }$ from *G*30:**end for**31:return S,C;

#### 4.3.5. Acceptance Criteria

Given a solution *S* and a new solution new_S generated from *S*, we define the difference of cost between new_S and *S* as Δ, as shown in Equation (12).

(12) Δ=calCost(new_S)−calCost(S)

In Equation (12), calCost(new_S) and calCost(S) represent the cost function value of the new generated solution and current solution respectively. If Δ<0, new_S is accepted as a better solution. Otherwise, it will be adopted according to the probability *p* defined in Equation (13).

(13) p=exp(−Δ/T)

It can be seen that a higher temperature has a higher probability to accept a worse solution as a new solution. However, in the end of the cooling process, only better solution will be reserved as a new solution, and it finally converges on the best solution.

#### 4.3.6. Stopping Condition

We define a temperature $T_{fin}$ to form a stopping condition. When the temperature is lower than $T_{fin}$, our algorithm will terminate.

## 5. Algorithm for ESPPWC Problem

As discussed in the above section, GMEDS and SAMEDS are proposed to solve the ESPPOC problem where edge sever vendors can customize the capacities of edge servers according to the historical statistic information. Unfortunately, these two algorithms are improper when there are constraints on the capacities of edge servers. To this end, we design a new Greedy-based Minimum Extended Dominating Set algorithm With Capacity constraint (GMEDSWC) to solve the ESPPWC problem. Similar to GMEDS, we apply *i*-tier dominating set to divide WMAN into disjoint clusters in GMEDSWC. The main difference between ESPPOC and ESPPWC is that there are constraints on the capacities of edge servers in ESPPWC problem.

Algorithm 3 gives the description of GMEDSWC in detail. In each iteration, for the the largest coverage of APs within *i* hops, we first choose an AP *s* with the largest value of extDeg(v,i) as the head of a cluster from the uncovered APs, as can be found in Lines 5–12. Then, we examine whether the cluster head *s* can satisfy computation demands of all the APs in the cluster. If the condition is satisfied, a cluster *S* composed *v* and the APs covered by it will be built, and the APs in *S* will be labeled as “covered” and deleted from the graph G. Otherwise, we iteratively remove a AP from the APs covered by *s* until all computation demands of *s* and APs covered by *s* is not greater than the capacity of the edge server co-located with *s*. In this solution, there are three strategies to rebuild the clusters: (1) big first; (2) random; and (3) small first. As the names suggest, big first strategy removes the AP with the highest computation demand, random strategy removes the AP randomly and small first strategy removes the AP with the lowest computation demand. The impact of different strategies on algorithm performance is shown in experimental results. After the cluster satisfying the computation demand constraint is built, it is then adjusted to meet the degree constraint and cluster size constraint as GMEDS does. Similarly, APs in the rebuilt cluster will be labeled as “covered” and deleted from G. Our algorithm will terminate when all APs are covered.

**Algorithm 3** Greedy-based minimum extended dominating set algorithm with capacity constraint.**Input:** A graph of the WMAN *G*, the tier of DS *i*, the capacity of edge servers, *c*, $D_{q}$, $S_{q}$; **Output:** Locations of edge servers, $D^{i}$, clusters of APs, *C*;
1:U←V; /* *V* is set of nodes in *G*, *U* is the set of nodes uncovered by $D^{i}$ */2:C←∅; /* *C* is set of the eventual clusters */3:$D^{i}\leftarrow \emptyset ;$ /* $D^{i}$ is the locations of edge servers */4:**while***U***do**5:    $s\leftarrow v_{1};$ /* $v_{1}$ is the first node in *U* */6:    **for** each v∈U **do**7:        compute the distance between *v* and all other vertexes in *U*, denoted by d(v,u)8:        compute the number of the nodes that d(v,u) is no more than *i*, denoted by extDeg(v,i)9:        **if** extDeg(v,i)>extDeg(s,i) **then**10:           s←v;11:        **end if**12:    **end for**13:    S←s∪extNei(s,i);14:    sumR←015:    **for** each v∈S **do**16:        sumR←sumR+r(v)17:    **end for**18:    **while** sumR>c **do**19:        remove a node from *S* by specific strategy, for instance $v^{\prime }$20:        $sumR\leftarrow sumR-r(v^{\prime })$21:    **end while**22:    new_cluster.head←s;23:    new_cluster.nodes←extNei(s,i);24:    **if** there exists a node *v* in *S* whose deg(v) is bigger than $D_{q}$ **then**25:        adjust the cluster until all the nodes satisfy the $D_{q}$ constraint26:    **end if**27:    **if** |S|>$S_{q}$ **then**28:        adjust the cluster until |S| < $S_{q}$29:    **end if**30:    C←C∪new_cluster;31:    $D^{i}\leftarrow D^{i}\cup s$;32:    remove the nodes in *S* from *G*;33:    U←U−S;34:**end while**35:return $D^{i},C$;

## 6. An Example for ESPPOC and ESPPWC Problems

**Figure 3:**
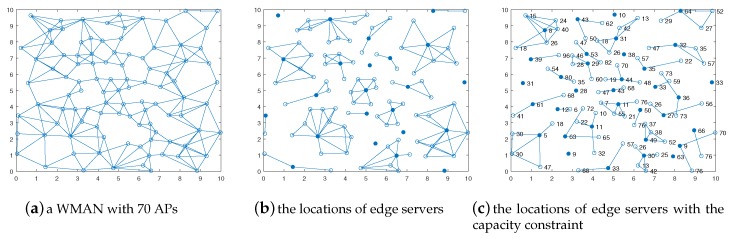
An example for ESPPOC and ESPPWC problems.

We simulated a WMAN, and in this section show an example for the solutions of ESPPOC and ESPPWC problem. As shown in Figure 3a, an area of 10 × 10 with 100 nodes was modeled as a city of 10 km × 10 km with 100 APs where there is an obvious city center. Moreover, APs were denser in the center than in the outskirts, which can represent the actual situation. It was supposed that a MEC service vendor is planning to deploy edge servers to serve the entire population of this city. The delay constraint was one hop and the degree constraint and cluster size constraint were omitted for simplicity. The basic requirement of this MEC service vendor is minimizing the number of edge servers due to the budget reason. If the capacities of edge servers can be configured, it is obviously a ESPPOC problem and it can be solved by computing the minimum one-tier dominating set. Thus, GMEDS was applied to solve this problem and the result is shown in Figure 3b. In the figure, solid circles represent the APs co-located with an edge server, namely cluster heads, and hollow circles represent the APs themselves. As shown in Figure 3b, the WMAN is divided into 23 clusters and all the in the WMAN can connect to one cluster head within one hop. This means the MEC service vendor only needs to deploy 23 edge servers to provide the service of the whole city and achieve the delay constraint simultaneously. In addition, if there are constraints on the capacities of edge servers, it is a ESPPWC problem. We could utilize GMEDSWC to solve it, and the result is shown in Figure 3c. In Figure 3c (refer to [14]), the numbers near APs are the computation demands of themselves which are generated randomly from 2500 MHz to 100,000 MHz. For simplicity, the numbers of computation demands were reduced 1000 times and the units of these numbers were omitted. In addition, the capacities of edge servers were identical, 200,000 MHz. We obtained that the WMAN is divided into 34 clusters and the sum of all the computation demands in each cluster is no greater than 200. It is worth noting that both results imply that the distribution of edge servers is consistent with the fact that the need of edge servers is more intense in the center than in the outskirts. Moreover, the constraints on capacities of edge servers will lead to the increase of the number of edge servers and the change of the locations of edge servers.

## 7. Experimental Analysis

We conducted extensive experiments to evaluate the proposed algorithms by synthetic network topologies where all algorithms ran on a machine with the Windows 10 and 64 bit operating system, 4 GHz Intel i7 CPU and 16 GB RAM. We should note that all proposed algorithms were implemented in Matlab.

### 7.1. Random Topology Generation

In our experiments, we generated synthetic topologies to model the real WMANs. Different from [14], we created an area to model the city, and took the the nodes as the APs. It was supposed that all APs have the same communication distance γ, and there exists a link between two APs if the distance between them is less than γ. In addition, let δ represent the minimum distance between two APs, which refers to the fact that two APs cannot be too close. Thus, the distance between the connected two nodes is greater than δ and less than γ. Given a random area of Mkm×Mkm, the number of APs *N*, γ and δ, we could generate the random topology step by step. First, we created an AP at a random location in the area and we regarded it as the center of the city. Then, new APs were added into the area and all the distances between the APs and the center were nearly exponentially distributed, which means APs in the area close to the center are denser than APs in other area [39]. To control the density of APs, two conditions must be met when new AP joins the area. One is that the AP must be in the communication coverage of an existing AP, and the other is the distance between the new AP and any existing AP cannot be less than δ. Moreover, to achieve the characteristic that the density of center is higher than elsewhere, when the nodes added to the area reach 70% of all nodes, we doubled the values of δ and γ. Finally, the random topology and its adjacency matrix were generated. Referring to [14], which denotes r(i) as the computing resource demand of AP $v_{i}$, we set r(i) as a variable from 2500 MHz to 100,000 MHz. For more realism, the capacity of each edge server was always bigger than 100,000 MHz. In the experiments, the related parameters are shown in Table 1, and we set *M*, δ and γ as 30, 0.5 and 1, respectively. Without loss of generality, we ran every experiment 100 times and the average results were used to report the performance.

### 7.2. Performance Evaluation of Algorithms for ESPPOC

For the largest search space, the initial temperature $T_{0}$ in SAMEDS was set to 10,000, and the stopping condition $T_{fin}$ was 0.01. In this way, the temperature was always suitable to all kinds of search spaces and the work to adjust the temperature for different network sizes could be neglected. Moreover, the cooling factor α was given a value of 0.99, which is in the suggested range (0.8–0.99). For comparison, we adopted the Random-ESPPOC algorithm as the benchmark algorithm, which randomly selects an AP to deploy an edge server until the whole network is covered.

**Figure 4:**
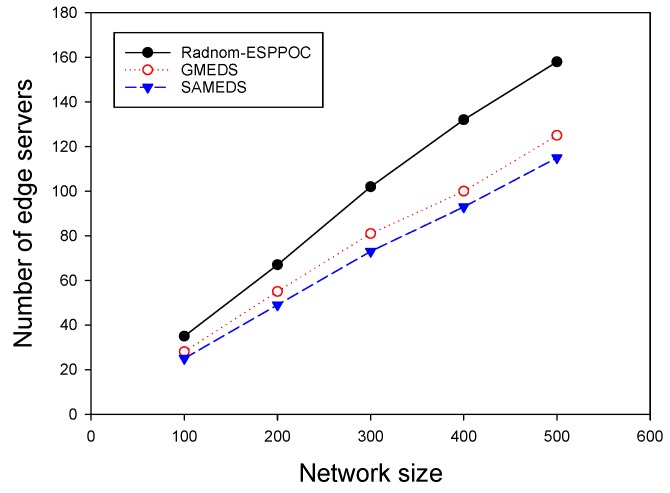
The impact of network size with on-demand capacities.

We evaluated the performance of different algorithms by varying the number of APs (*N* = 100–500), while fixing the delay constraint at 1. Figure 4 plots the curves of the number of edge servers of different algorithms, in which it can be seen that all three curves have a growth trend as the network size increases. Compared with Random-ESPPOC, GMEDS and SAMEDS 20.6% and 27.5% fewer edge servers on average, respectively.

**Figure 5:**
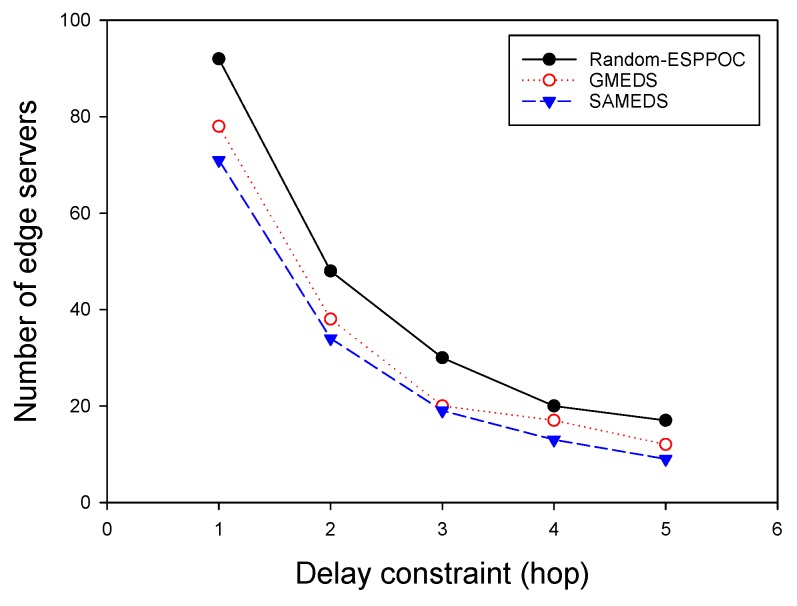
The impact of delay constraint with on-demand capacities.

Then, we evaluated the performance of these algorithms by varying the delay constraint from one hop to five hops while fixing the network size at 300. As shown in Figure 5, as the delay constraint increases, the number of the edge servers will decrease for all algorithms, and both GMEDS and SAMEDS have better performance than Random-ESPPOC. Compared with Random-ESPPOC, GMEDS and SAMEDS have 20.3% and 29.5% fewer edge servers on average, respectively. Both results imply the economy and the great potential of our solutions in real world.

### 7.3. Performance Evaluation of Algorithms for ESPPWC

**Figure 6:**
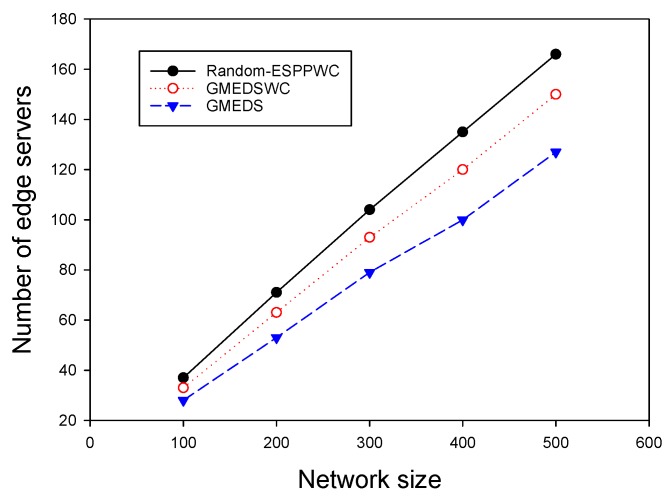
The impact of network size with identical capacities.

**Figure 7:**
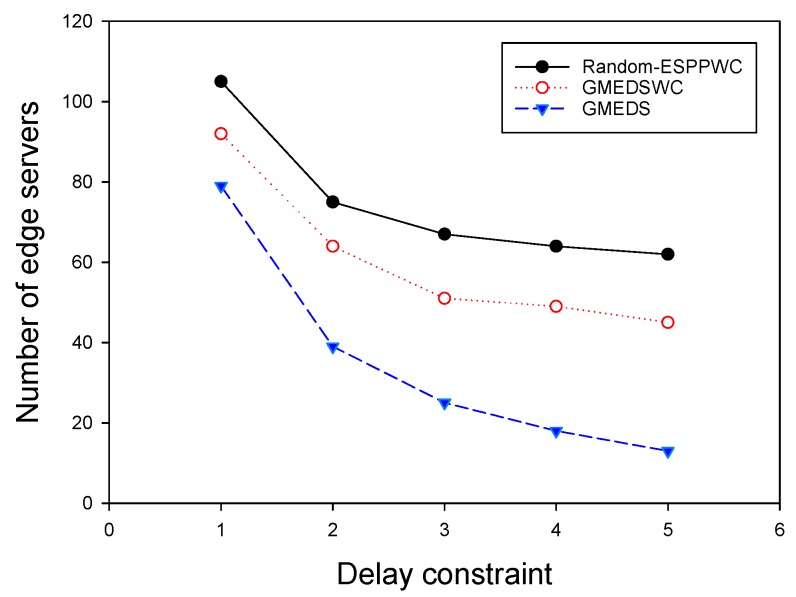
The impact of delay constraint with identical capacities.

We investigated the algorithm for ESPPWC. Similar to the results presented in the last subsection, we first chose Random-ESPPWC as the benchmark algorithm to compare with GMEDSWC. The difference between Random-ESPPOC and Random-ESPPWC is that Random-ESPPWC should satisfy the given constraints. In addition, to show the influence of the capacity on the placement solutions, we compared GMEDSWC with GMEDS in the same topologies. It is worth noting that the mentioned big first strategy was adopted to achieve the best performance of GMEDSWC, and the capacity of each edge server was given a value of 200,000 MHz. We first evaluated these three algorithms by varying the network size and fixing delay constraint, as above. Figure 6 shows that, with the increase of network size, the number of edge servers grows, as is consistent with the realistic condition. It can be seen that GMEDSWC and GMEDS outperform Random-ESPPOC, having 10.5% and 24.6% fewer edge servers, respectively. Figure 6 also shows that the capacity constraint results in the increase of the need of edge servers. As shown in Figure 7, the number of edge servers decreases as the delay constraint increases, and about 49.0% more edge servers are required when the capacity constraint is attached.

### 7.4. Impact of Important Parameters on Performance of ESPPWC

**Figure 8:**
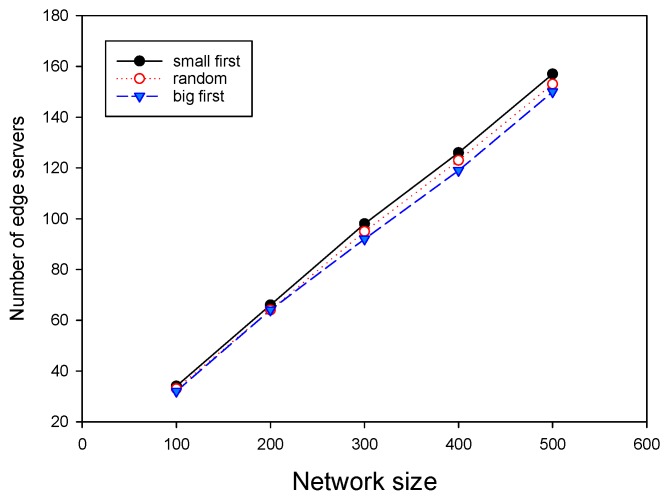
The impact of cluster rebuilding strategy on the number of edge servers.

**Figure 9:**
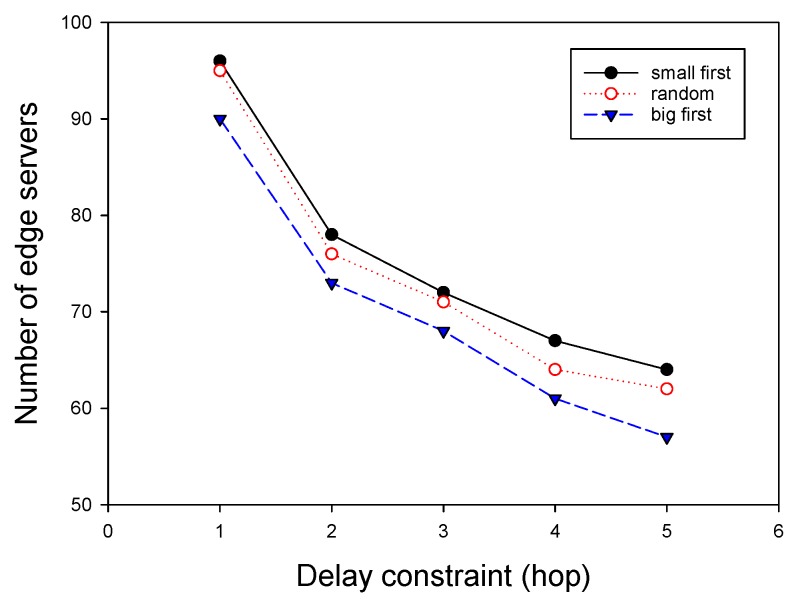
The impact of cluster rebuilding strategy on the number of edge servers.

**Figure 10:**
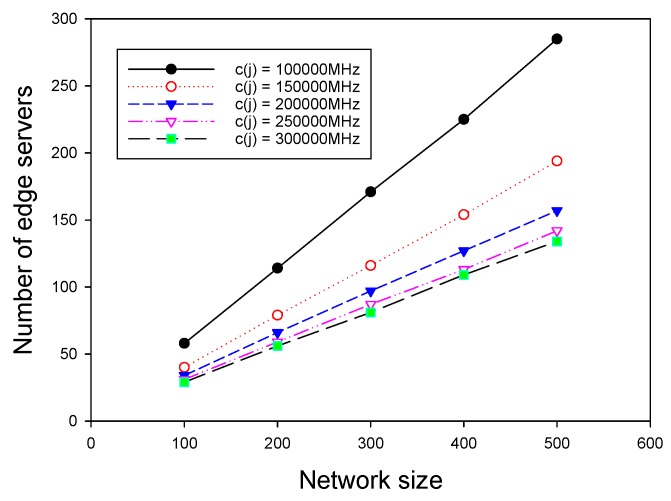
The impact of capacity on the number of edge servers.

**Figure 11:**
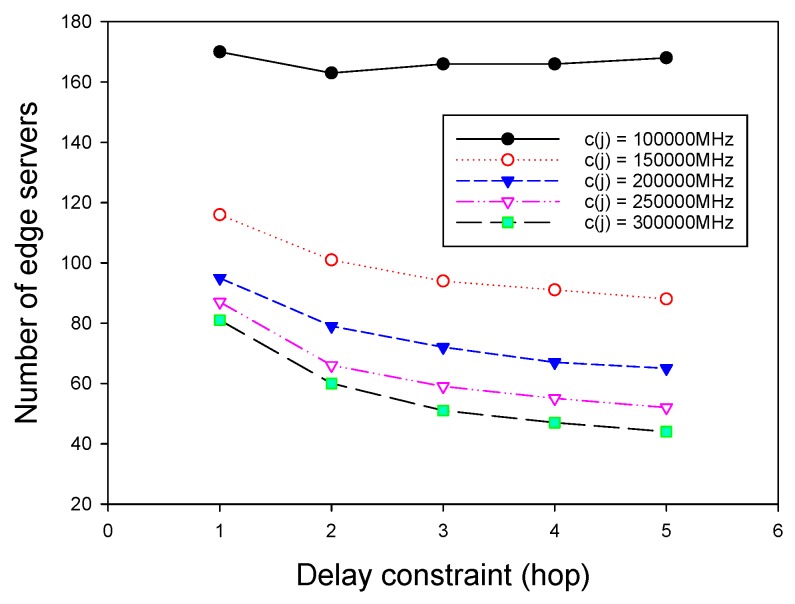
The impact of capacity on the number of edge servers.

We finally investigated the impact of two important parameters on the performance of GMEDSWC. As described in Section 5, if the sum of computation demands in a cluster exceed the given capacity of an edge server, there are three strategies to rebuild the cluster, and different cluster rebuilding strategies have different results. Therefore, it is nontrivial to investigate the influence of different rebuilding strategies on the performance of ESPPWC. In addition, it is interesting to study the effects of different capacities of edge servers on the performance of ESPPWC. As shown in Figure 8 and Figure 9, big first strategy has the best performance and small first always achieves the worst performance. Therefore, it can be seen that choosing a proper cluster rebuilding strategy is also an important issue. Figure 10 and Figure 11 show that the value of capacities has great effects on the performance of GMEDSWC, namely the higher the capacity is, the fewer edge servers are needed. In Figure 10, we can see that, when the capacity of edge server is set to 300,000 MHz, the number of edge servers is 66.0% less than the situation with the capacity set to 100,000 MHz. Moreover, in Figure 11, an additional 108.6% edge servers are needed when the capacity of edge server is decreased from 300,000 MHz to 100,000 MHz.

### 7.5. Impact of Degree Constraint on Performance of Algorithms

**Figure 12:**
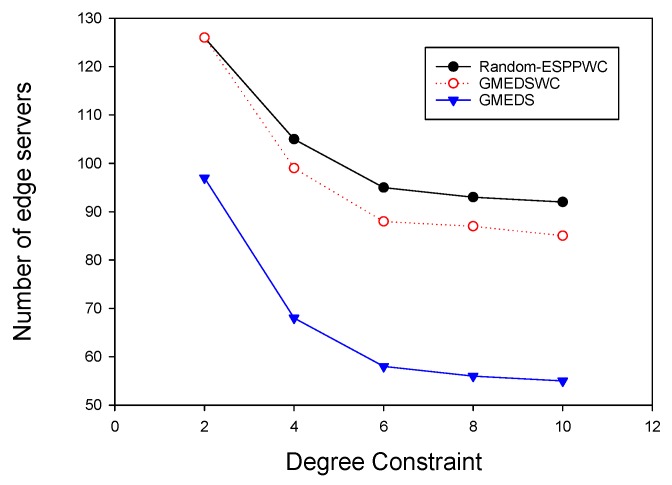
The impact of cluster degree constraint on the number of edge servers.

We analyzed the impact of degree constraint $D_{q}$ on the number of edge servers by fixing the network size at 300 and delay constraint at three hops. The cluster size constraint was relaxed to eliminate the interference. As shown in Figure 12, both GMEDSWC and GMEDS are significantly affected by the degree constraint where the number of edge servers decreases as the degree constraint increases. This is because every AP can connect more APs when the degree constraint increases. In addition, we can also see that both curves have a gentle trend, which indicates that, when the degree constraint reaches a specific value (here the value is 6), its impact will be weakened.

### 7.6. Impact of Cluster Size Constraint on Performance of Algorithms

**Figure 13:**
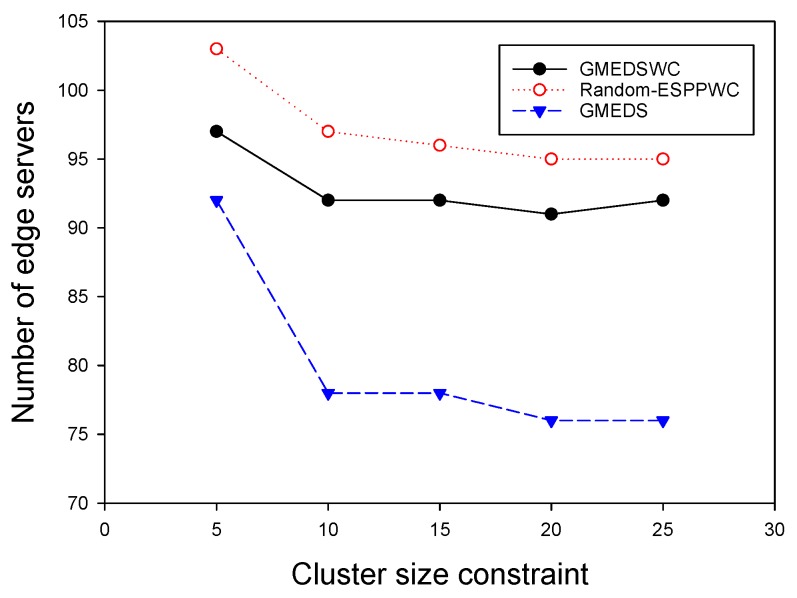
The impact of cluster size on the number of edge servers.

We finally investigated the impact of cluster size constraint $S_{q}$ on the performance of our algorithms. Similar to the last subsection, the degree constraint was neglected and we evaluated the performance of these algorithms by fixing network size at 300 and delay constraint at three hops. Figure 13 shows that the number of edge servers obtained by all three algorithms decreases as $S_{q}$ increases because each edge server will have more chances to cover as many APs as possible. Likewise, the curves have same gentle tails, from which we can know that, when $S_{q}$ increases to a specific value, its influence on the performance of our algorithms will fade.

## 8. Conclusions and Future Work

There is only a small amount of research on the edge server placement problem in WMAN and most of them ignore the budget limitation of MEC service vendors. In this paper, we focus on saving the cost of deploying edge servers in a WMAN, and we address it as the problem of minimizing the number of edge servers while ensuring some constraints related to network QoS. Then, we present an ILP formulation for the edge server placement problem and divide it into two categories of problems according to the capacity constraint. Due to the poor scalability of the ILP, we extend the definition of dominating set in graph theory and transform the problem into the minimum *i*-tier dominating set problem. Furthermore, we propose two heuristic algorithms and a greedy based algorithm to solve these two kinds of problems, respectively. The simulation results show that the proposed algorithms are promising. For our future work, it is interesting to investigate how to use other existing networks, such as 4G network, to deploy edge servers efficiently.

## Figures and Tables

**Table 1:** Default experiment settings.

Parameter	Value
M	30
δ	0.5
γ	1
iter	2000
$T_{0}$	10,000
$T_{fin}$	0.01
α	0.99
